# The association between the C-reactive protein-triglyceride glucose index and myocardial injury after acute ischemic stroke: a machine learning analysis of the brain-heart axis

**DOI:** 10.3389/fcvm.2026.1742657

**Published:** 2026-05-21

**Authors:** Huijuan Pu, Guoping Zhao, Dailing Yao, Yumin Wang, Ni An, Yan Liu, Chenyang Li, Xiuli Zhang, Jie Liu, Wanling Wu, Hong Zhu, Lei Li, Defeng Pan

**Affiliations:** 1 Department of General Practice, The Affiliated Hospital of Xuzhou Medical University, Xuzhou, China; 2 Department of Cardiology, The Affiliated Hospital of Xuzhou Medical University, Xuzhou, China; 3 Department of Geriatric Medicine, The Affiliated Hospital of Xuzhou Medical University, Xuzhou, China

**Keywords:** acute ischemic stroke, brain-heart axis, c-Reactive protein-Triglyceride glucose index, machine learning, myocardial injury

## Abstract

**Background and purpose:**

Myocardial injury is a common and serious complication following acute ischemic stroke (AIS), that contributes to poor functional outcomes. The C-reactive protein-triglyceride glucose index (CTI), a novel composite marker reflecting inflammation and insulin resistance (IR), has shown prognostic value in cardio-cerebrovascular diseases. This study used machine learning (ML) for feature selection to investigate the association between the CTI and myocardial injury following AIS.

**Methods:**

A retrospective cohort study was conducted, in which patients with AIS within 72 h of symptom onset were enrolled. The primary endpoint was myocardial injury after AIS. The CTI was calculated via the formula 0.412 × ln [Hs-CRP (mg/L)] + ln [TG (mg/dL) × FBG (mg/dL) / 2]. Four ML algorithms were applied to identify predictive variables. Multivariate logistic regression and restricted cubic spline (RCS) analyses were used to assess the independent associations and dose-response relationships between the CTI and myocardial injury.

**Results:**

Among the 842 patients, 288 (34.2%) experienced myocardial injury. The CTI was significantly greater in the myocardial injury group (*P* < 0.01). The ML models consistently identified the CTI as the top predictor. Multivariate analysis revealed that the CTI was independently associated with myocardial injury (*OR* = 2.61, 95% *CI*: 1.99–3.42). RCS analysis revealed a positive linear relationship (*P* for nonlinea*r* = 0.443). Compared with the TyG index (*AUC* = 0.596) and Hs-CRP (*AUC* = 0.689) alone, the CTI demonstrated moderate discriminative ability and showed improved performance (*AUC* = 0.713).

**Conclusions:**

The CTI was significantly associated with myocardial injury and its integration of IR and inflammatory status suggests that it may serve as a moderately discriminative indicator for risk stratification.

## 1 Introduction

Acute ischemic stroke (AIS) remains a leading cause of mortality and long-term disability worldwide. It is projected that by 2030, the global number of deaths and disabilities due to stroke will reach 22 million (1, 2). Although the widespread application of reperfusion therapies, such as intravenous thrombolysis and endovascular thrombectomy, has significantly improved the short-term survival rate for AIS patients, outcomes after AIS remain influenced by various complications, among which cardiovascular involvement plays a pivotal role.

The brain-heart axis refers to a bidirectional neurocardiac regulatory network through which acute cerebral injury affects cardiac structure and function via hypothalamic-pituitary-adrenal (HPA) axis, the systemic inflammatory response, sympathetic overexcitation, and insulin resistance (IR) (3, 4). Dysregulation of this axis following AIS gives rise to a spectrum of cardiac manifestations collectively termed stroke-heart syndrome (SHS) (5). SHS encompasses myocardial injury, arrhythmias, heart failure (HF), myocardial infarction (MI) and Takotsubo syndrome (5, 6). Notably, the presence of SHS is closely associated with adverse functional outcomes in patients and has become a significant risk factor for early mortality after stroke, ranking as the second leading cause of death, particularly within the first few weeks after stroke onset (7).

Among the manifestations of SHS, myocardial injury typically identified by elevated cardiac troponin (cTn) levels represents one of the most frequent and clinically relevant phenotypes. However, in most stroke patients, elevated cTn is not accompanied by typical symptoms of myocardial ischemia or electrocardiogram changes, making them prone to clinical oversight (8). As a specific biomarker for myocardial injury, cTn enables the early identification of subclinical myocardial injury, and its elevation is positively correlated with stroke severity and mortality risk (8). On this basis, the American Heart Association/American Stroke Association (AHA/ASA) guidelines explicitly recommend routine cTn testing for all suspected stroke patients upon hospital admission to assess the degree of myocardial injury. These findings provide a critical basis for developing early intervention strategies, reducing the incidence of cardiovascular complications, and improving patient prognosis (9).

IR and chronic inflammation have been confirmed as key pathophysiological basis of cardio-cerebrovascular diseases (10, 11). The triglyceride-glucose index (TyG), a validated surrogate marker of IR, has demonstrated significant clinical value in predicting stroke, cardiovascular events, and metabolic related diseases (11–13). Similarly, high-sensitivity C-reactive protein (hs-CRP), a marker of systemic inflammation, correlates with stroke severity and unfavorable prognosis (14). In recent years, researchers have proposed combining inflammatory and IR to allow a more comprehensive assessment of cardio-cerebrovascular risk. The C-reactive protein-triglyceride glucose index (CTI) is a novel composite index that emerged from this context, and is designed to collectively reflect the body's inflammatory status and IR level. Previous studies have demonstrated its prognostic value in cancer mortality, incidence of cardiovascular disease, and stroke risk in hypertensive or general populations (15–17). Nevertheless, evidence regarding the relationship between CTI andmyocardial injury following AIS remains lacking.

Traditional statistical methods often face limitations, including variable selection bias, model overfitting, and insufficient predictive accuracy, when handling clinical data characterized by high dimensionality, nonlinearity, multicollinearity, and complex interactions. In contrast, machine learning (ML) approaches can automatically identify complex nonlinear relationships among variables, uncover potential interactions, and enhance the predictive accuracy and generalizability of models. In recent years, ML approaches have demonstrated strong potential in predicting stroke outcomes, stratifying cardiovascular event risk, and identifying complications (18, 19).

Thus, this study aimed to investigate the association between CTI and post-AIS myocardial injury by applying ML algorithms for feature selection to identify key variables associated with the outcome.

## 2 Methods

### 2.1 Study population and design

This retrospective cohort study utilized data from the electronic medical records system of the Affiliated Hospital of Xuzhou Medical University. The study protocol was approved by the hospital's Ethics Committee (Approval No.XYFY2025-KL008-01). The study cohort comprised patients admitted between January 2021 and December 2023 with a diagnosis of AIS, which was established in accordance with the 2019 guidelines (20).

The inclusion criteria were as follows: (1) AIS confirmed by neuroimaging (CT or MRI); (2) hospital admission within 72 h of onset; (3) completion of a cardiac troponin T (cTnT) test at admission; and (4) if cTnT was elevated, a repeat test was required within 48 h.

**Figure 1:**
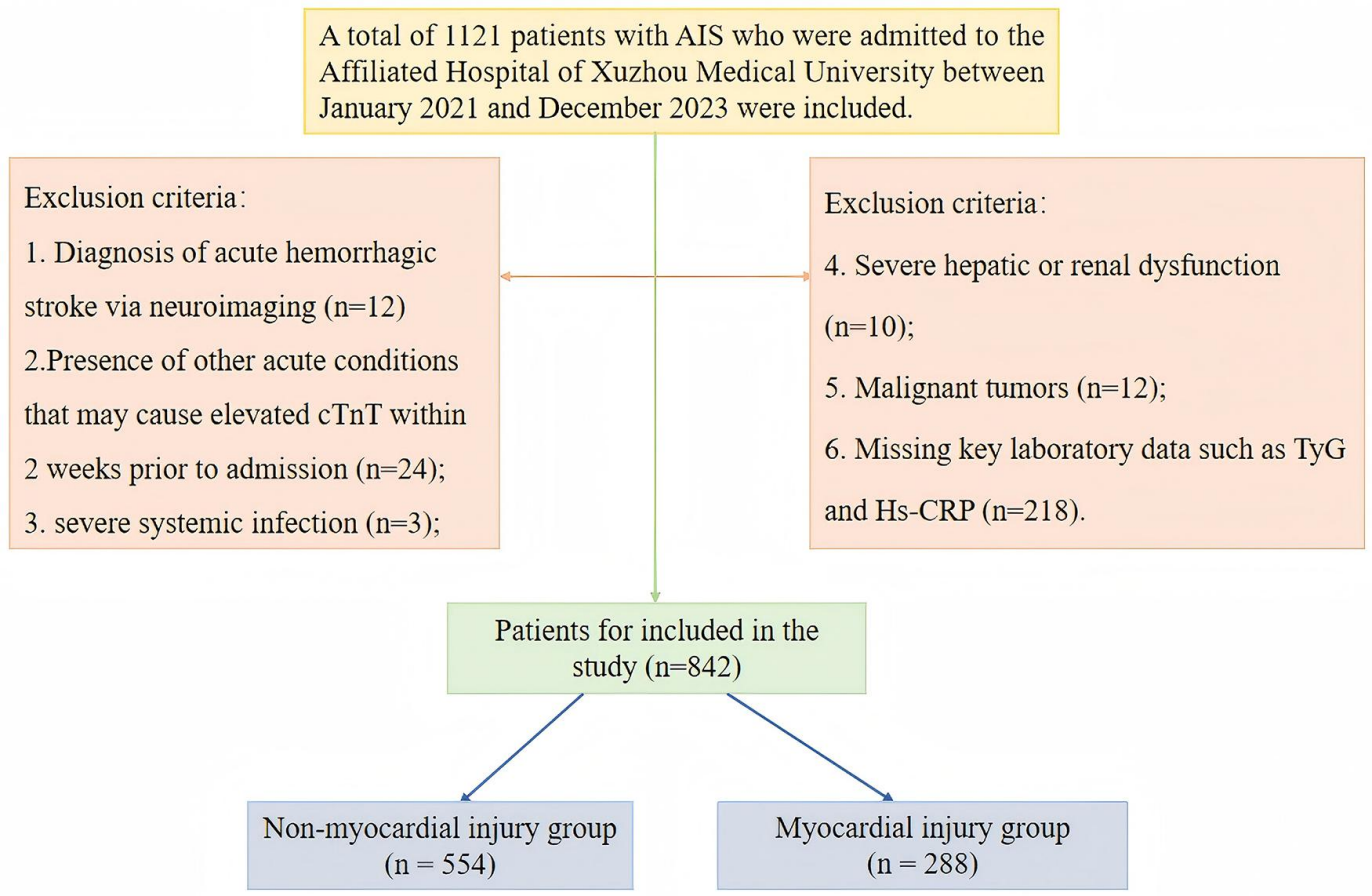
Study enrollment flowchart.

The exclusion criteria included the following: (1) acute hemorrhagic stroke confirmed by neuroimaging (21); (2) other acute conditions that could cause elevated cTnT within two weeks prior to admission, such as acute myocardial infarction, HF, major cardiac surgery, sepsis, acute kidney injury, rhabdomyolysis, pulmonary embolism, or infective endocarditis; (3) concomitant severe systemic infection; (4) severe hepatic or renal insufficiency (Child-Pugh class C, MELD score ≥15, eGFR <15 mL/min/1.73 m^2^, or receiving dialysis); (5) malignant tumors; and (6) missing key variables such as TyG and Hs-CRP (Figure 1).

### 2.2 Data collection

The collected data includes the following categories:

- Demographic data: Age, gender, body mass index (BMI).
- Cardiovascular risk factors: hypertension, diabetes mellitus (DM), smoking history, and drinking history.
- Comorbidities: coronary heart disease (CHD), prior stroke, and atrial fibrillation (AF).
- Medication history: including antiplatelet agents, anticoagulants, beta-blockers, and statins.
- Stroke treatment modalities: traditional drugs, intravenous thrombolysis, and mechanical thrombectomy.
- Neurological status assessment: Stroke severity was assessed via the National Institutes of Health Stroke Scale (NIHSS) and Glasgow Coma Scale (GCS) scores (22). The location of insular cortical ischemic lesions was evaluated via diffusion-weighted magnetic resonance imaging (DWI).
- Laboratory parameters: relevant biochemical and hematological parameters.
- Calculation of TyG and CTI:
  TyG index = ln [Triglyceride (TG)(mg/dL) × Fasting blood glucose (FBG)(mg/dL)/2];
  CTI = 0.412 × ln [Hs-CRP (mg/L)] + ln [TG (mg/dL) × FBG (mg/dL)/2].

### 2.3 Outcome event

The primary outcome was post-AIS myocardial injury, defined according to the Fourth Universal Definition of Myocardial Infarction. Cardiac troponin T (cTnT) was measured using a high-sensitivity assay (Elecsys hs-cTnT, Roche Diagnostics), with a 99th percentile upper reference limit of 14 ng/L. Sex-specific thresholds were not applied. Acute myocardial injury was defined as a rise and/or fall in cTnT levels of >20%, with at least one value above the 99th percentile upper reference limit (23–25) Clinical information, including symptoms, electrocardiographic findings, and echocardiography, was reviewed to support outcome adjudication.

### 2.4 Statistical analysis

Statistical analyses were performed via SPSS 27.0 and R Studio 4.3.1. *P*-value < 0.05 was considered statistically significant.

#### 2.4.1 Analysis of baseline characteristics

Normally distributed continuous variables are presented as the means ± standard deviations ($\bar{x}\pm s$) and were compared via the independent samples *t*-test. Nonnormally distributed continuous variables are expressed as medians (M) with interquartile range (IQR) M (P25, P75) and were compared via the Mann–Whitney *U*-test. Categorical variables are summarized as frequencies and percentages (%), and group comparisons were made via the chi-square test or Fisher's exact test, as appropriate.

#### 2.4.2 Machine learning and feature selection

ML algorithms were employed to evaluate and rank the importance of the included variables. Four representative ML methods were selected: least absolute shrinkage and selection operator (LASSO) regression, gradient boosting machine (GBM), eXtreme gradient boosting (XGBoost), and random forest (RF). To enhance model reliability and reproducibility, all algorithms underwent systematic hyperparameter tuning and validation: LASSO used 10-fold cross-validation to determine the regularization parameter, GBM and XGBoost were implemented via the caret framework with repeated cross-validation for parameter optimization (using 10-fold cross-validation), and RF was internally validated using out-of-bag error estimation. The use of these four distinct methods for feature selection helps mitigate potential bias inherent to any single algorithm and increases the likelihood of identifying stable and robust predictors. The consensus feature variables identified by all four methods were considered the most stable set, and the selection process was visualized via Venn and Sankey diagrams.

#### 2.4.3 Multivariate logistic regression

The consensus variables derived from the ML feature selection were incorporated into a multivariate logistic regression model to assess their independent associations with myocardial injury. Confounding factors were adjusted based on clinical relevance and previous literature. Variables that remained statistically significant after adjustment were retained in the final model. The results are presented as odds ratios (OR) with corresponding 95% confidence intervals (CI).

#### 2.4.4 Restricted cubic spline (RCS) analysis

Restricted cubic splines (RCS) were used to examine the potential dose-response relationship between the CTI and the risk of myocardial injury. The significance of the nonlinear relationship was tested.

#### 2.4.5 Performance evaluation

The discriminative ability of the CTI, TyG index, and Hs-CRP for post-stroke myocardial injury was assessed by constructing receiver operating characteristic (ROC) curves and calculating the area under the curve (AUC). The DeLong test was used to compare the AUCs of CTI with those of the TyG index and Hs-CRP to evaluate whether CTI provided improved discriminative ability.

Furthermore, net reclassification improvement (NRI) analysis was performed to evaluate the incremental value of CTI. A baseline model including conventional clinical variables was constructed, and CTI was subsequently added to this model. The NRI was calculated to assess the improvement in risk classification provided by the addition of CTI.

## 3 Results

### 3.1 Population characteristics

A total of 842 patients with AIS were included in this study, of whom 288 (34.2%) developed myocardial injury. Compared with those in the non-myocardial injury group, patients in the myocardial injury group were older; had higher NIHSS scores; had lower GCS scores; and had a significantly greater prevalence of DM, AF, and CHD.

In terms of laboratory parameters, the myocardial injury group presented significantly elevated levels of NT-terminal pro-brain natriuretic peptide (NT-proBNP), creatine kinase isoenzyme (CK-MB), FBG, neutrophil count, Hs-CRP, serum creatinine (Scr), blood urea nitrogen (BUN), cystatin C (CysC), D-dimer, and fibrinogen (FIB). In contrast, the levels of hemoglobin (Hb), lymphocyte count, total cholesterol (TC), low-density lipoprotein cholesterol (LDL-C), estimated glomerular filtration rate (eGFR), and albumin (ALB) were significantly lower. Most importantly, both the TyG index and CTI were significantly greater in the myocardial injury group than in the non-myocardial injury group. Furthermore, the incidence of myocardial injury increases significantly with increasing CTI (Table 1).

**Table 1:** Baseline characteristics of the non-myocardial injury group and myocardial injury group.

Variables	Non-myocardial injury group	Myocardial injury group	*P Value*
	(*n* = 554)	(*n* = 288)	
Demographic data			
Age (yr)	65.00 (56.00, 72.00)	72.00 (65.00, 78.00)	<0.01
BMI (kg/m^2^)	24.97 (22.84, 27.34)	24.18 (22.22, 26.69)	0.01
Gender *n* (%)			0.50
Female	178 (32.13)	86 (29.86)	
Male	376 (67.87)	202 (70.14)	
Cardiovascular risk factors *n* (%)			
Hypertension	374 (67.51)	204 (70.83)	0.32
Diabetes Mellitus	194 (35.02)	139 (48.26)	<0.01
Smoking	268 (48.38)	157 (54.51)	0.09
Drinking	331 (59.75)	172 (59.72)	0.99
Comorbidities *n* (%)			
AF	22 (3.97)	47 (16.32)	<0.01
CHD	64 (11.55)	51 (17.71)	0.01
Prior Stroke	157 (28.34)	89 (30.90)	0.44
Medication history *n* (%)			
Antiplatelet drugs	195 (35.20)	122 (42.36)	0.04
Anticoagulant drugs	19 (3.43)	35 (12.15)	<0.01
Beta-blockers	99 (17.87)	77 (26.74)	<0.01
Statins	188 (33.94)	121 (42.01)	0.02
Therapy methods *n* (%)			<0.01
Traditional drugs	478 (86.28)	212 (73.61)	
Thrombolysis	56 (10.11)	56 (19.44)	
Thrombectomy	20 (3.61)	20 (6.94)	
Neurological status			
Insular cortical lesions *n* (%)	53 (9.57)	62 (21.53)	<0.01
NIHSS Score	1.00 (0.00, 2.00)	2.00 (1.00, 5.00)	<0.01
GCS	15.00 (14.00, 15.00)	14.00 (13.00, 15.00)	<0.01
Laboratory parameters			
NT-proBNP (pg/mL)	128.00 (58.48, 348.50)	248.00 (88.20, 1,140.00)	<0.01
CK (U/L)	71.00 (52.00, 103.00)	73.00 (49.00, 117.00)	0.31
CK-MB (ng/mL)	1.45 (1.10, 2.04)	1.75 (1.20, 2.54)	<0.01
Hb (g/L)	139.00 (128.00, 150.00)	135.00 (123.00, 147.00)	<0.01
* N* (10^9^/L)	4.08 (3.25, 5.20)	4.37 (3.42, 5.88)	<0.01
L (10^9^/L)	1.50 (1.20, 1.90)	1.40 (1.10, 1.72)	<0.01
PLT (10^9^/L)	207.50 (176.00, 251.00)	205.00 (168.75, 245.25)	0.11
FBG (mmol/L)	5.30 (4.76, 6.56)	6.48 (5.14, 8.70)	<0.01
HbA1c (%)	6.00 (5.60, 7.30)	6.20 (5.70, 7.70)	0.06
TC (mmol/L)	4.29 (3.61, 5.09)	4.03 (3.34, 4.86)	<0.01
TG (mmol/L)	1.40 (1.07, 1.92)	1.44 (1.09, 2.17)	0.25
LDL-C (mmol/L)	2.52 (1.93, 3.12)	2.22 (1.66, 2.83)	<0.01
HDL-C (mmol/L)	0.97 (0.83, 1.16)	0.98 (0.83, 1.18)	0.85
LDH (U/L)	177.00 (160.00, 204.00)	183.50 (161.75, 211.25)	0.06
Scr (umo/L)	59.00 (49.00, 70.00)	62.50 (52.00, 75.00)	<0.01
eGFR (mL/min)	120.00 (99.11, 120.00)	108.98 (87.90, 120.00)	<0.01
BUN (mmol/L)	5.09 (4.27, 6.33)	5.56 (4.51, 6.89)	<0.01
UA (umo/L)	282.50 (232.00, 341.00)	285.00 (233.00, 329.25)	0.88
CysC (mg/L)	0.91 (0.80, 1.05)	1.01 (0.88, 1.13)	<0.01
ALB (g/L)	41.40 (39.02, 43.80)	40.00 (37.58, 42.10)	<0.01
AST (U/L)	18.00 (15.00, 23.00)	17.00 (15.00, 21.00)	0.30
ALT (U/L)	17.00 (11.00, 25.00)	15.00 (11.00, 21.00)	0.007
D-dimer (mg/L)	0.29 (0.19, 0.55)	0.51 (0.27, 1.67)	<0.01
FIB (g/L)	2.74 (2.35, 3.26)	2.90 (2.49, 3.62)	<0.01
APTT (sec)	27.00 (25.33, 29.17)	27.10 (25.00, 28.80)	0.42
PT (sec)	10.60 (10.00, 11.20)	10.70 (10.10, 11.30)	0.35
TT (sec)	16.10 (15.40, 16.90)	15.90 (15.28, 16.80)	0.07
HCY (umo/L)	15.96 (12.86, 19.72)	16.21 (13.55, 20.57)	0.13
Hs-CRP (mg/L)	1.55 (0.50, 3.40)	4.00 (1.40, 21.02)	<0.01
TyG index	8.74 (8.38, 9.13)	8.98 (8.48, 9.53)	<0.01
CTI			<0.01
≤4.62	179 (32.31)	32 (11.11)	
4.63–5.07	153 (27.62)	57 (19.79)	
5.07–5.63	134 (24.19)	76 (26.39)	
≥5.64	88 (15.88)	123 (42.71)	

BMI, body mass index; AF, atrial fibrillation; CHD, coronary heart disease; NIHSS, National Institutes of Health Stroke Scale; NT-proBNP, brain natriuretic peptide; CK, creatine kinase; CK-MB, creatine kinase isoenzyme; Hb, hemoglobin; N, neutrophil; L, lymphocyte; PLT, platelet; FBG, fasting blood glucose; HbA1c, glycosylated hemoglobin; TC, total cholesterol; TG, triglyceride; LDL-C, low-density lipoprotein cholesterol; HDL-C, high-density lipoprotein cholesterol; LDH, lactic dehydrogenase; Scr, serum creatinine; eGFR, estimated glomerular filtration rate; BUN, blood urea nitrogen; UA, uric acid; CysC, cystatin C, ALB, albumin; AST, aspartate aminotransferase; ALT, alanine aminotransferase; FIB, fibrinogen; APTT, activated partial thromboplastin time; PT, prothrombin time; TT, thrombin time; HCY, homocysteine; Hs-CRP, high-sensitivity C-reactive protein; TyG, triglyceride-glucose Index; CTI, C-reactive protein-triglyceride glucose index.

### 3.2 Feature variable selection

**Figure 2:**
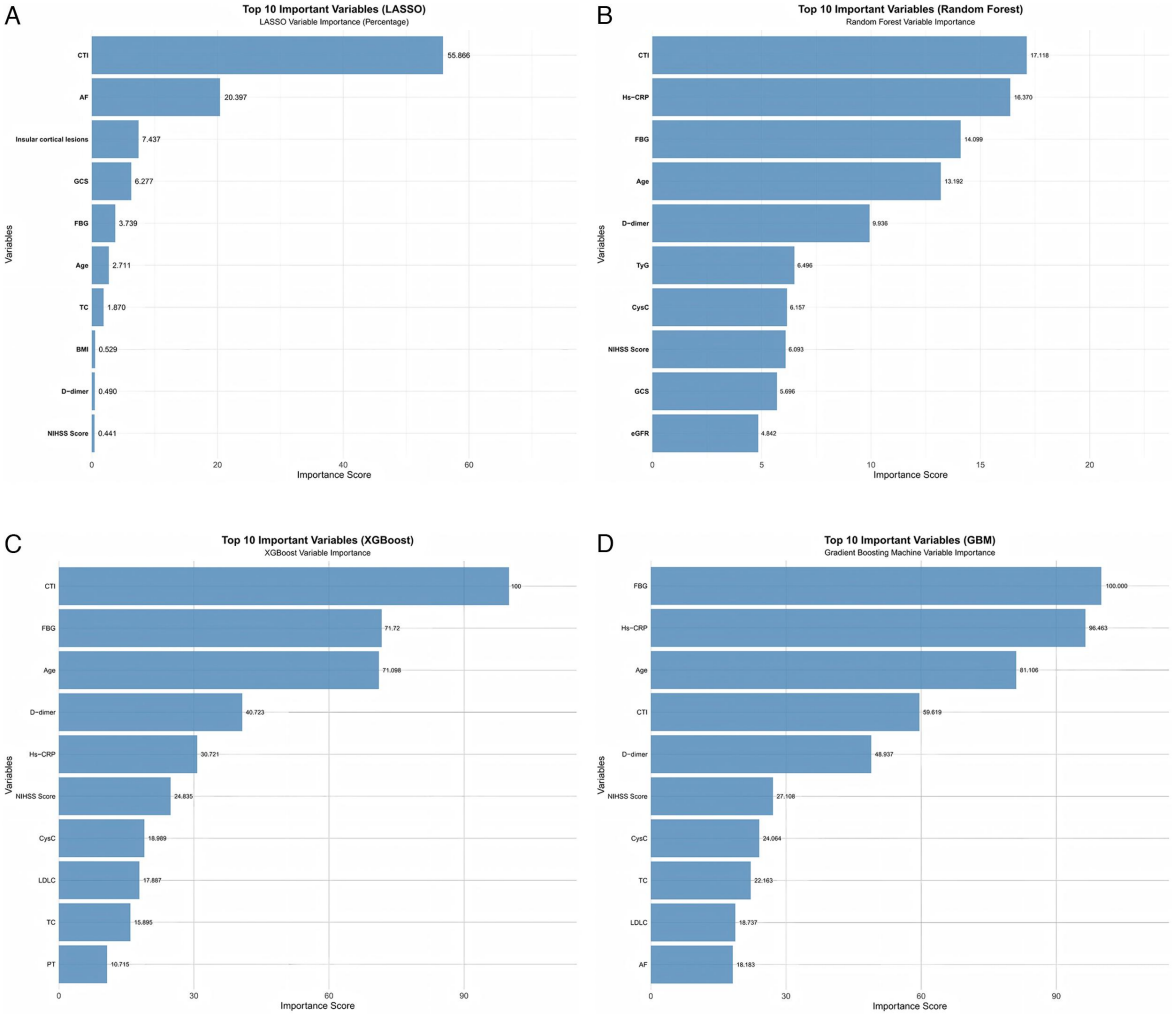
Top 10 most important features in each machine learning model. **(A)** LASSO regression. **(B)** Random Forest. **(C)** XGBoost. **(D)** GBM.

The selection of feature variables was performed via four machine learning algorithms. Figure 2 displays the top 10 most important variables identified by each model. Throughout the feature selection process employing four distinct machine learning methods, CTI emerged as a consistently key variable.

**Figure 3:**
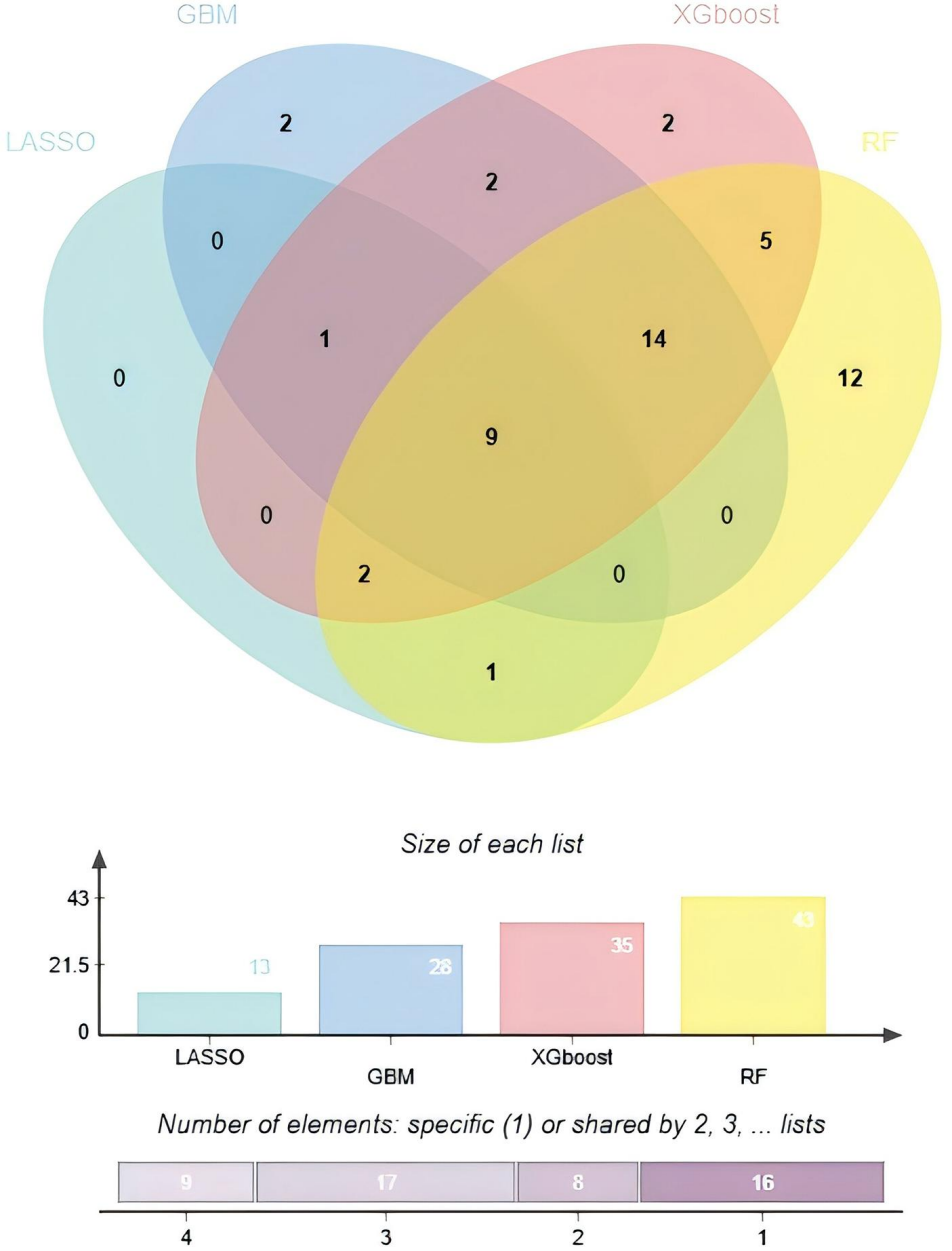
Venn diagram analyzes the results of four machine learning model.

To synthesize the results from the different algorithms and enhance the robustness of the feature selection, we took the intersection of all non-zero variables selected by the four methods via a Venn diagram. This process yielded a final set of nine consensus variables: age, BMI, AF, NIHSS score, TC, FBG, eGFR, D-dimer, and CTI (Figure 3).

**Figure 4:**
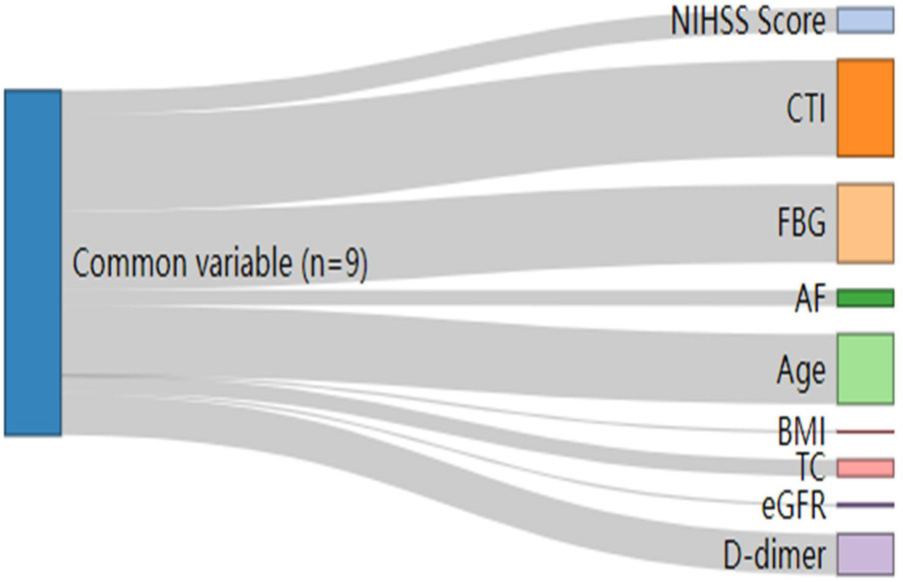
Sankey diagram visualizing variable importance.

The core predictive variables were visualized in a Sankey diagram, with link width representing their relative importance. This visualization clearly illustrates the flow and contribution of variables from the initial set to the final selection, and show that CTI is the most important variable (Figure 4).

Multivariate logistic regression analysis was conducted to assess the independent associations of the nine candidate variables with myocardial injury. After adjustment for confounders, age, NIHSS score, BMI, TC, eGFR, FBG, and CTI remained independently associated with myocardial injury (*P* < 0.05). D-dimer, however, was not statistically significant (*P* = 0.124) and was excluded from the final model (Table 2).

**Table 2:** Multivariate logistic regression analysis for post-AIS myocardial injury.

Variables	*β*	*S.E*	*Z*	*P*	*OR* (95%CI)
AF	0.67	0.33	2.06	0.04	1.95 (1.03–3.69)
NIHSS Score	0.04	0.02	2.48	0.013	1.04 (1.01–1.08)
Age	0.05	0.01	5.86	<0.01	1.05 (1.03–1.07)
BMI	−0.07	0.03	−2.87	<0.01	0.93 (0.88–0.98)
TC	−0.22	0.08	−2.87	<0.01	0.80 (0.68–0.93)
D-dimer	0.07	0.05	1.54	0.12	1.07 (0.98–1.18)
eGFR	−0.01	0.00	−3.41	<0.01	0.99 (0.98–0.99)
FBG	0.12	0.04	3.26	<0.01	1.12 (1.05–1.21)
CTI	0.96	0.14	6.94	<0.01	2.61 (1.99–3.42)

AF, atrial fibrillation; NIHSS, National Institutes of Health Stroke Scale; TC, total cholesterol; eGFR, estimated glomerular filtration rate; FBG, fasting blood glucose; CTI, C-reactive protein-triglyceride glucose index.

The BMI and TC were negatively correlated with myocardial injury, which may appear counterintuitive to traditional cardiovascular risk paradigms. Further age-stratified analyses revealed that the inverse association between BMI and myocardial injury was primarily observed in patients aged ≥65 years, whereas no significant association was detected in patients aged <65 years. In contrast, TC was not significantly associated with myocardial injury in either age subgroup (Supplementary Table S1).

#### 3.3 Associations between the CTI and myocardial injury after AIS

**Figure 5:**
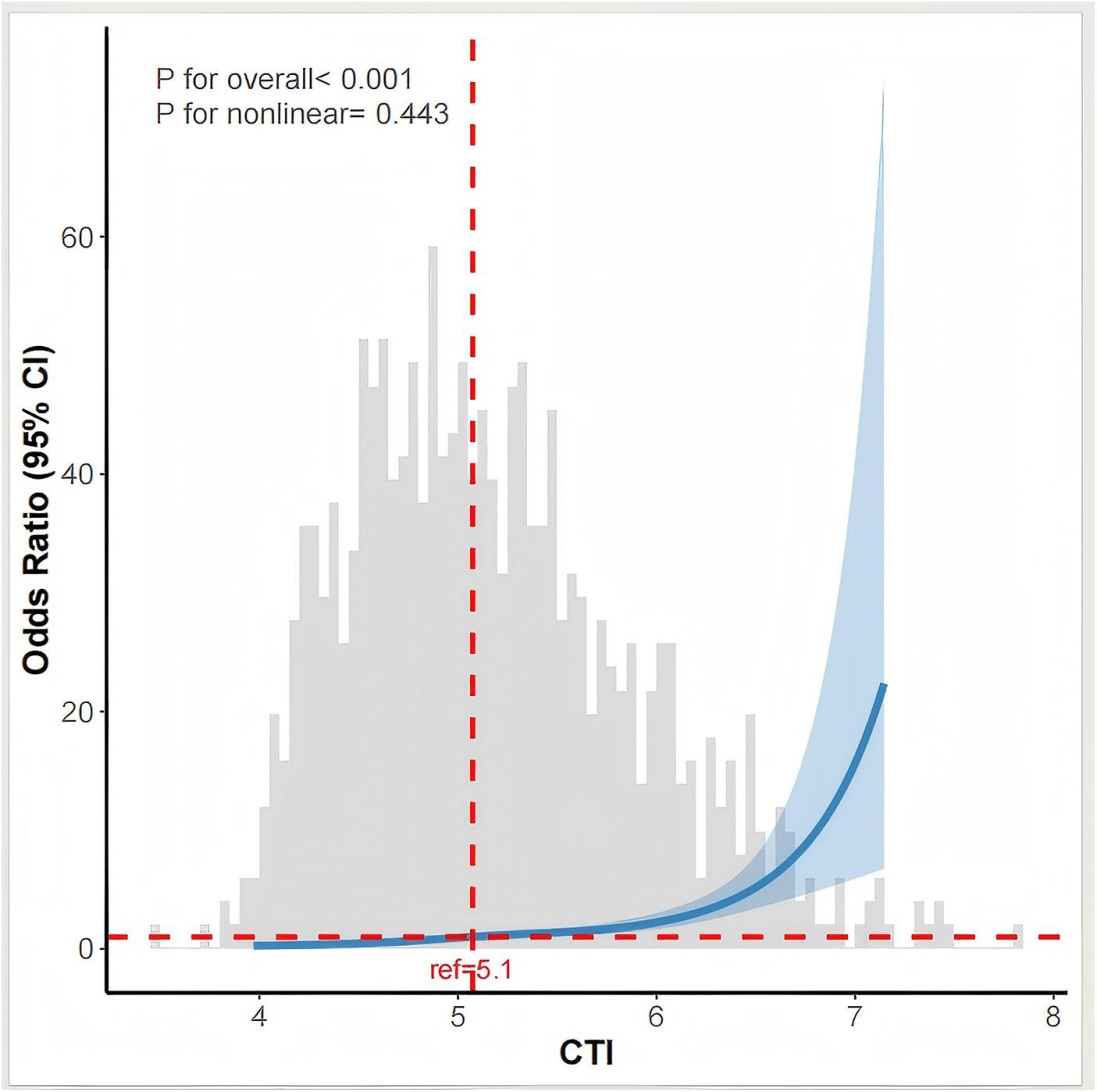
Association between the CTI and myocardial injury.

**Figure 6:**
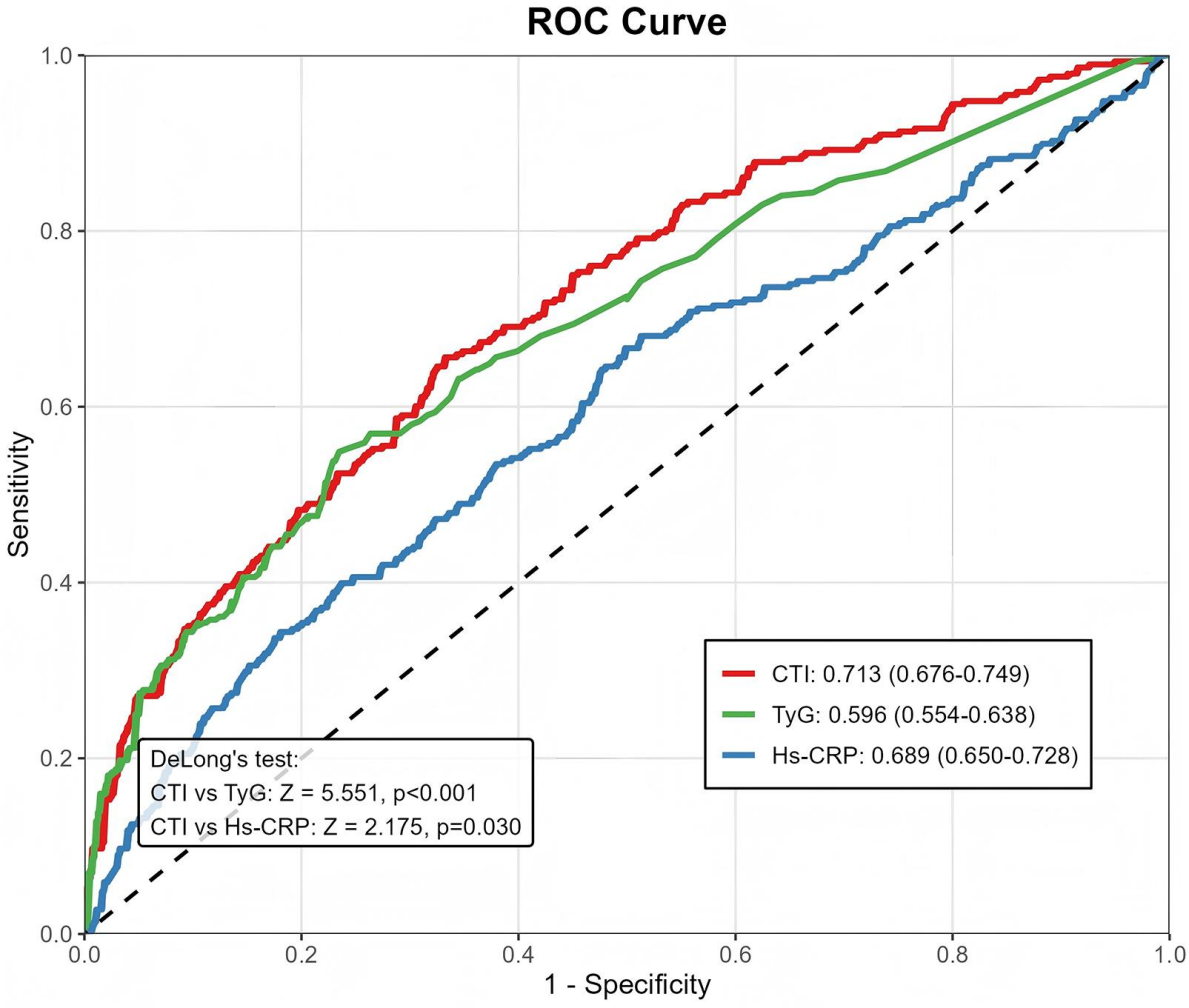
ROC curves and DeLong's test.

RCS analysis revealed a linear positive correlation between CTI and the risk of poststroke myocardial injury (*P* for overall < 0.001; P for nonlinear = 0.443), indicating that the risk of myocardial injury increased continuously with increasing CTI (Figure 5). ROC curve analysis showed that the AUC of CTI for predicting post-stroke myocardial injury was 0.713 (95% CI: 0.676–0.749), compared with 0.596 (95% CI: 0.554–0.638) for the TyG index and 0.689 (95% CI: 0.650–0.728) for hs-CRP. Pairwise comparisons using DeLong's test confirmed that CTI had significantly better predictive performance than TyG (Z = 5.551, *P* < 0.001) and hs-CRP (Z = 2.175, *P* = 0.030) (Figure 6). NRI analysis further demonstrated that the addition of CTI to the baseline model improved patient risk classification, with an NRI of 0.42 (95% CI: 0.28–0.56).

## 4 Discussion

This study is the first to systematically investigate the association between the CTI and myocardial injury after AIS via ML algorithms. The main findings are as follows: (1) the CTI was significantly associated with post-AIS myocardial injury and showed a positive linear dose-response relationship; (2) using four distinct machine learning algorithms, nine consensus predictive variables were identified, among which the CTI demonstrated the highest importance; and (3) the association strength of CTI with post-stroke myocardial injury was greater than that of the TyG index or hs-CRP when evaluated individually. Taken together, these findings support the relevance of CTI in the context of inflammatory and IR associated with myocardial injury after AIS.

Notably, inverse associations between BMI and myocardial injury were observed in the primary analyses, which may appear counterintuitive to traditional cardiovascular risk paradigms.

Several potential explanations may account for these observations. First, lower BMI and TC levels in patients with AIS, particularly in older individuals, may reflect frailty, malnutrition, or reduced physiological reserve rather than a protective effect. Second, reverse causation is possible, as more severe illness or systemic stress may lead to decreased lipid levels and body mass. Third, treatment-related factors, such as statin use or acute-phase metabolic changes, may influence TC levels. Finally, residual confounding from unmeasured or incompletely measured variables cannot be excluded. In age-stratified analysis, the inverse association between BMI and myocardial injury was primarily observed in patients aged ≥65 years, whereas no significant association was found in younger patients, supporting the hypothesis that BMI may serve as a proxy for frailty or nutritional status in older populations (26, 27). In contrast, the association between TC and myocardial injury was not consistent across age-stratified analyses, suggesting limited robustness. Therefore, these findings should not be interpreted as protective effects of higher BMI or TC levels in the context of myocardial injury after AIS.

As a composite index integrating both inflammation and IR, CTI likely provides a more comprehensive reflection of the patient's metabolic and immune status compared with a single biomarker. Compared with a single biomarker, the CTI demonstrated greater predictive accuracy for myocardial injury, suggesting its potential application value in the early identification of SHS.

Previous studies have established that both the TyG index and Hs-CRP are independently associated with the risk of cardiovascular events after stroke (7, 14). Ruan et al. first proposed the CTI and demonstrated its strong prognostic ability in cancer patients and the general population (15). In recent years, the application of CTI in cardio-cerebrovascular diseases has gained increasing attention. A study by Huo et al. based on the CHARLS database revealed a linear positive correlation between CTI and stroke risk, which remained consistent across different glycemic statuses (28). Furthermore, Ma et al. indicated that persistently elevated CTI levels were significantly associated with an increased risk of cardiovascular disease (CVD) in middle-aged and older adults (29). Building upon this existing evidence, our study expands the clinical application scenario of the CTI by being the first to confirm its independent association with acute myocardial injury following AIS, which exhibits a linear dose-response relationship.

The application of ML algorithms provided a robust feature selection strategy for this study. A cross-validated consensus from four distinct algorithms LASSO, XGBoost, GBM, and RF identified nine core variables strongly associated with myocardial injury. The CTI consistently demonstrated high feature importance across all the models, reinforcing its stability and reliability as a key predictor (18, 19). This finding was further corroborated by multivariate logistic regression, which confirmed that the CTI was independent associated with myocardial injury (OR = 2.61, 95% CI: 1.99–3.42), highlighting its potential utility in clinical risk assessment.

### Limitations

Several limitations of this study should be acknowledged. First, as a single-center retrospective study, our findings may be subject to selection bias, and their generalizability requires further validation. In addition, although multiple clinically relevant variables were adjusted for, residual confounding cannot be entirely excluded. Second, this study focused solely on myocardial injury occurring during the acute phase of AIS (within 48 h of admission) and did not include follow-up data on long-term cardiovascular outcomes, which limits the assessment of the prognostic value of CTI beyond the acute stage. Third, CTI was calculated based on baseline laboratory measurements at admission. Dynamic changes in inflammatory and metabolic markers during hospitalization were not analyzed, and thus the potential impact of longitudinal CTI trajectories on myocardial injury could not be explored.

### Future directions

Future research should include the following directions. First, multicenter prospective cohort studies to validate the predictive value of CTI across diverse populations and ethnic groups. Second, incorporation of long-term follow-up to evaluate the relationship between CTI and cardiovascular events after stroke. Third, investigating dynamic changes in CTI during the post-stroke period may provide further insights into its potential role in monitoring disease progression and therapeutic response.

## 5 Conclusions

In conclusion, CTI was independently associated with myocardial injury after acute ischemic stroke. Compared with traditional single biomarkers, CTI showed improved discriminatory ability. The observed positive linear relationship further highlights its potential value for early risk stratification in patients with AIS.

## Data Availability

The raw data supporting the conclusions of this article will be made available by the authors, without undue reservation.
