# Supplementary material for: The association between the C-reactive protein-triglyceride glucose index and myocardial injury after acute ischemic stroke: a machine learning analysis of the brain-heart axis
Source: Front Cardiovasc Med. 2026 May 21;13:1742657. doi: 10.3389/fcvm.2026.1742657 (PMC13233255; doi:10.3389/fcvm.2026.1742657)
Supplement: Supplementary file 1 [file Table1.docx]

Supplementary Table S1 stratification analysis

|  | ***OR* (95%CI)** | ***P-Value*** |
| --- | --- | --- |
| **BMI** |  |  |
| **<65 (yr)** | 0.94 (0.86-1.03) | 0.173 |
| **≥65 (yr)** | 0.93 (0.88-0.98) | **0.004** |
| **TC** |  |  |
| **<65 (yr)** | 0.94 (0.74-1.18) | 0.602 |
| **≥65y (yr)** | 0.90 (.076-1.07) | 0.239 |

Abbreviations: BMI: body mass index;TC: total cholesterol.
